# Beyond the Immune System: The Role of Resident Cells in Asthma and COPD

**DOI:** 10.1155/2012/968039

**Published:** 2012-05-17

**Authors:** Peter Borger, Brian Oliver, Irene Heijink, Georgia Hardavella

**Affiliations:** ^1^Pulmonary Cell Research, Department of Biomedicine, University Hospital Basel, Basel, Switzerland; ^2^Woolcock Institute of Medical Research, The University of Sydney, Sydney, NSW, Australia; ^3^Departments of Pathology & Medical Biology and Pulmonology, University Medical Center Groningen, Groningen, The Netherlands; ^4^Department of Thoracic Medicine, University College London Hospital, London, UK

Asthma and chronic obstructive pulmonary disease (COPD) are the two most prominent chronic inflammatory lung diseases, and their prevalence is still increasing. Although both diseases are associated with genetic and environmental factors, their precise etiologies are still unclear. Recent advances in biology and medicine have introduced new technologies to study the genetics and function of resident cells of the lungs, and the data demonstrate a much broader range of impact than previously envisaged. The function of resident cells of the lung may be crucial to fully understand the pathology of asthma and COPD. Knowledge and understanding of resident cells will lead to the development of improved animal models, more successful therapies, novel tools to characterize asthma, and COPD and provide better care to patients. In our present special issue, several outstanding research groups present their original research articles that will stimulate the continuing efforts to understand the molecular pathology underlying asthma and COPD, the development of strategies to treat these conditions, and the evaluation of outcomes. Moreover, several important novel insights on the role of resident cells in asthma and COPD are being reviewed in this special issue.

Y. Matsumura reviews the proteinase activity of many common allergens and shows their interaction with lung epithelial cells; they induce disruption of the tight junctions between epithelial cells, activate the protease-activated receptor-2, and lead to the production of thymic stromal lymphopoietin. Hence, allergen source-derived proteases are a potentially critical factor in the development of allergic sensitization and appear to be strongly associated with heightened allergenicity. S. Kolahian and R. Gosens describe a novel role of the parasympathetic neurotransmitter acetylcholine in the regulation of airway remodeling and inflammation in respiratory disease. Recent data have indicateed that nonneuronal cells, including smooth muscle cells, secrete acetylcholine and express receptors for acetylcholine. S. Kolahian and R. Gosens focus on the role of acetylcholine in smooth muscle cell function and review data to show that the activation of acetylcholine receptors in these cells can lead to proliferation, production of growth factors, inflammatory mediators, and deposition of extra-cellular matrix proteins. Moreover, it can lead to increased smooth muscle mass, contractility, mucus gland remodeling and airway inflammation *in vivo* in a guinea pig model of asthma. Similarly, tiotropium studies show that acetylcholine may contribute to cigarette smoke-induced neutrophilic airway inflammation in a murine model of COPD. In addition to the new role of acetylcholine, N. Miglino et al. present data on another novel target to reduce bronchial smooth muscle remodeling in their original research article. They show that calreticulin, which has been described as a negative regulator of C/EBP*α* [1], is also able to reduce airway smooth muscle (ASM) cell proliferation and may thus provide beneficial effects on airway remodeling. This pathway was insensitive to corticosteroids, which may explain why airway remodeling in asthma patients is refractory to corticosteroid therapy [2]. In particular subpopulations of asthma patients do not respond well to the current therapy available for asthma. To identify more effective treatments, it is of importance to unlock the underlying mechanisms and understand the genetic background. A. Faiz and K. Burgess review the history of expression microarray technologies, that is, genome-wide association studies (GWASs)/locus fine mapping, gene candidate approaches, and gene expression studies (gene-expression microarrays, including the Gene-Chip), and their contribution to increase our understanding of asthma pathology. For instance, GWASs have been essential in the discovery of many asthma-associated genes, including disintegrin and metalloproteinase domain-containing protein 33 (ADAM33). In addition, the 3′ expression arrays have shown that treatment with IL-13 caused deregulation of a number of asthma-related genes in different cell types of the airway, including smooth muscle cells and airway epithelium.

The ASM cell has been recognized as a critical effecter cell in the pathophysiology of asthma for almost a century. The interplay between the smooth muscle and the pathophysiology of asthma is reviewed by A. Ozier and colleagues. Specifically they discuss the mechanisms driving airway hyper-responsiveness in asthma, such as mediator release, altered excitation/contraction coupling, and the role of airway smooth muscle in bronchial inflammation and remodeling. T. Chiba et al. prove a compelling review on the role of the arylhydrocarbon receptor (AhR), a nuclear receptor which responds to dioxins and dioxin-like compounds in cigarette smoke and environmental pollutants. In their review they discuss the role of AhR in asthma and COPD, focusing upon how AhR modulates the immunological responses in allergic and inflammatory diseases such as bronchitis, asthma, and chronic obstructive pulmonary disease (COPD) and the crosstalk of AhR signaling with other ligand-activated transcription factors such as peroxisome proliferator-activated receptors (PPARs). In addition to the role of mesenchymal cells in innate immunity, mesenchymal cells also have the ability to direct and in-turn can be influenced by the adaptive immune system. One novel example of this interaction is the role of immunoglobulin receptors found upon the surface of mesenchymal cells. In this issue, N. Roth reviews the role of immunoglobulin receptors upon mesenchymal cell function and importantly addresses some of the basic questions in this field: why do tissue-forming cells express immunoglobulin receptors and do tissue-forming cells process immunoglobulin receptor-bound particles?

Several drugs are currently in use targeting the inflammatory component of the asthmatic disorder, including steroids and beta-mimetics [3]. It is well-established that rhinovirus (RV)-induced asthma exacerbations account for high asthma-related health costs and morbidity [4]. D. van Ly and coworkers studied the molecular mechanism underlying this pathology involving RV-induced nuclear factor kappa B (NF-*κ*B)-dependent inflammation. To establish the role of NF-*κ*B inhibitors in RV-induced IL-6 and IL-8 and RV replication, they used pharmacological inhibitors of NF-*κ*B, and steroids and/or *β*2 agonists were used as comparison. Their data suggest that targeting NF-*κ*B alone is unlikely to be an effective treatment compared to current asthma therapeutics. Q. Ge and coworkers examined the effect of combined corticosteroids and long acting beta2-agonists on *in vitro* extracellular matrix protein production by TGF-stimulated airway smooth muscle (ASM) cells present in isolated bronchial rings of nonasthmatic individuals. They conclude that current combination asthma therapies are unable to prevent or reverse remodeling events that are regulated by ASM cells. Current asthma therapies have also been in the spotlight of J. C. Allen et al. who investigated corticosteroid insensitivity *in vitro* and were the first to examine the effect of dexamethasone on the mitogen PDGF-BB-induced cyclin D1 upregulation in ASM cells from both nonasthmatics and asthmatics. They showed that cyclin D1 mRNA and protein up-regulation in cells from asthmatic patients cannot be totally inhibited. These results agree with earlier evidence that corticosteroid-inhibited proliferation occurs only in ASM cells from nonasthmatics [5] and suggest that there are corticosteroid insensitive proliferative pathways in asthmatics that need to be further investigated towards new efficacious antiremodeling strategies.

A. Soltani and coworkers have had substantial experience in using Collagen IV antibody as a blood vessel marker in bronchial biopsies [6]. Therefore, they compared the utility of anti-Collagen IV and anti-Factor VIII antibodies as markers for blood vessels in bronchial biopsies from COPD versus normal subjects and investigated the differences in the vessels' profiles that they stained. They showed that anti-Collagen IV antibody tends to stain more vessels in the Rbm and bigger vessels overall in both the Rbm and LP, while anti-Factor VIII antibody stains relatively smaller vessels. A novel finding of this study was the increased leakiness of the vessels in the Rbm in current smokers with COPD which is ripe for speculation and further study and could be factored into the concepts of the pathogenesis of smoking-related airway disease pathophysiology. The complex pathophysiology of COPD and asthma can be clarified by the use of recent advances in CT imaging. G. Dournes et al. present a thorough yet concise review of quantitative measurements of airway wall and lung parenchyma in both COPD and asthma by using CT. Quantification of airway wall and lung parenchyma has demonstrated strong correlations with clinical, functional, and pathological features in humans or animal models.

Taken together, this special issue provides a focused overview and state-of-the-art science related to the role and function of resident cells of the lung, and how they may be involved in developing and maintaining asthma and COPD pathologies.

**Peter Borger** **Peter Borger** **Brian Oliver** **Brian Oliver** **Irene Heijink** **Irene Heijink** **Georgia Hardavella** **Georgia Hardavella**
